# Caregiver Input to Optimize the Design of a Pediatric Care Planning Guide for Rehabilitation: Descriptive Study

**DOI:** 10.2196/rehab.7566

**Published:** 2017-10-24

**Authors:** Mary A Khetani, Heather K Lim, Marya E Corden

**Affiliations:** ^1^ Children's Participation in Environment Research Lab Department of Occupational Therapy University of Illinois at Chicago Chicago, IL United States

**Keywords:** pediatrics, social participation, goals, patient-reported outcome, eHealth

## Abstract

**Background:**

Caregiver input has informed the design of a valid electronic patient-reported outcome (PRO) measure for use in pediatric rehabilitation. This proxy assessment may be further developed to expedite and enhance patient-centered care planning processes, but user input is first needed to finalize the core requirements that will guide its design.

**Objective:**

The objective of this study was to examine the feasibility of a stepwise process for building on a baseline assessment of young children's participation in activities to develop a care plan relevant to pediatric rehabilitation.

**Methods:**

A cross-sectional descriptive study design was employed using qualitative methods. Data were collected via Web-based technology and by telephone. Twenty-five caregivers of young children (9 with developmental delays, 16 without delays) and between 1 and 7 years were recruited from a subsample of parents who had previously enrolled in a Web-based validation of a PRO on children’s participation and provided consent for future contact. Each caregiver completed a demographic questionnaire and Young Children’s Participation and Environment Measure (YC-PEM) online, followed by a 20- to 60-min semistructured and audiotaped phone interview to review and build upon PRO results as summarized in an electronic report. Interview data were content coded to the interview guide and reviewed by multiple research staff to estimate feasibility according to stepwise completion rates, perceptions of difficulty in step completion, and perceptions of overall utility.

**Results:**

Half of the participants in the final study sample (N=25) fully completed a stepwise process of building on their baseline PRO assessment to develop an initial care plan for their child. In most cases, similar stepwise completion rates and trends in the approaches taken for step completion were found regardless of the child’s disability status. However, more parents of children with disabilities reported difficulties in rank ordering their priorities for change and identified child-focused strategies for goal attainment. Nearly 77% (19/25) of users were willing to use the process to develop and communicate intervention priorities and strategies with professionals, family, and friends.

**Conclusions:**

Results informed revisions to the care planning guide before usability and feasibility testing of an initial Web-based prototype that is now underway.

## Introduction

### Background

Pediatric occupational therapists typically play a direct role in helping children with developmental disabilities and delays to participate in activities of daily life [1]. They are key members of pediatric rehabilitation teams who strive to deliver evidence-based and tailored therapies targeting functional outcomes [2] so as to mitigate social disparities in rehabilitation service use [3]. Occupational therapists rely on self or proxy report to monitor a child’s participation as compared with observing the child’s performance of discrete tasks [4,5]. Hence, providers need access to valid and feasible patient-reported outcome (PRO) assessments to gather caregiver input about young children’s participation for planning and delivering care that is responsive to patient priorities [6].

Due to time and resource constraints, pediatric rehabilitation providers need efficient ways to gather PRO data. For example, service eligible families in early intervention [7] need to have a care plan that is developed within 45 days of referral and reflects family priorities. Semistructured and face-to-face interviews with parents and primary caregivers are not routinely completed because of time and resource constraints. For example, the Routines-Based Interview (RBI) takes up to 120 min and 2 trained providers to complete [8].

Advances in rehabilitation assessment and technology [9] may afford for valid and more feasible family assessment. For example, the Young Children’s Participation and Environment Measure (YC-PEM) is a newly developed electronic assessment of children’s participation. The YC-PEM content, scaling, and layout decisions were informed by caregiver input [10-12] and are intended to offer several user benefits, which are as follows: (1) comprehensive assessment of participation in home, school, and community settings; (2) assessing multiple dimensions of a child’s participation (frequency, involvement, change desired); (3) assessing for environmental impact on participation in each setting; and (4) Web-based format affording feasible self-administration. Initial psychometric evidence suggests that the Web-based YC-PEM provides valid, reliable, and feasible assessment of participation and environmental impact on participation among young children with and without developmental disabilities [13]. Validation has focused on establishing the validity of known-groups [14] and modeling environmental impact on participation when applied to younger children [15]. The YC-PEM is now a recognized common data element for studies involving children with cerebral palsy and other neurological disorders [16]. Culturally adapted versions will increase instrument uptake in clinical research contexts [17] and afford for additional psychometric validation.

Recent work has been undertaken to explore the utility of deploying the YC-PEM within an intervention context [18-20]. For individual families, YC-PEM results may be helpful as a springboard for collaborative care planning with providers, supporting patient-centered care. As YC-PEM assessment results will not automatically produce a viable care plan, caregivers will need to complete additional work to synthesize their assessment results to develop goals with focused and feasible action plans to improve their child’s participation. However, caregivers often manage this complex task of improving their child’s participation with limited or delayed intervention support while balancing competing time demands. For this reason, electronic health (eHealth) technologies that help caregivers organize a plan-of-care flexibly, on their own schedule, may enhance caregiver–provider collaboration during care-planning activities.

Participation and Environment Measure Plus (PEM+) is a guide that is compatible with YC-PEM and may expedite care plan development and strengthen patient’s engagement in discussions and decisions about their values, needs, and desires that shape meaningful care (ie, patient-centered care) [21]. Caregivers are expected to complete PEM+ to specify their priorities for change, generate goals for their child, and design initial intervention strategies for goal attainment. PEM+ was designed with caregiver and provider input [18-20].

### Objective

The purpose of this study was to examine the feasibility of a revised PEM+ prototype for use within an early childhood-care-planning context. Study results will guide assessment of whether to build and test PEM+ usability as an eHealth technology for use in rehabilitation.

## Methods

### Participants

**Figure 1:**
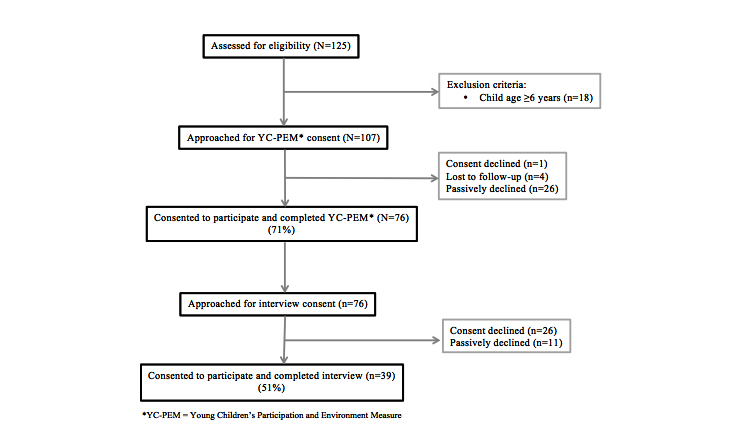
Enrollment Consort (Consolidated Standards of Reporting Trials) flow diagram.

This observational study employed a cross-sectional descriptive design. Data were drawn from a convenience sample of 125 caregivers of children with and without developmental disabilities and delays. They had initially consented to future contact during a Web-based YC-PEM validation study when their children were in the age group of 0 to 5 years (Time 1: June 2013-October 2013) and then enrolled online in a longitudinal cohort study 1 year later when their children were between the ages of 1 and 6 years (Time 2: October 2014-March 2015). At the Time 1 enrollment, all caregivers met the following inclusion criteria: (1) could read and write in English; (2) resided in the United States or Canada; (3) were 18 years or older; (4) were parents or legal guardians of a child aged between 0 and 5 years; and (5) had Internet access. For this study, a total of 76 caregivers enrolled in Time 2 data collection. These caregivers accessed a Web-based platform to consent to complete the YC-PEM online. After YC-PEM completion, 39 of these 76 caregivers also consented for a phone interview to discuss and build on their YC-PEM results using PEM+ (see Figure 1).

### Measures

#### Demographic Questionnaire

Caregivers were asked to report on family factors (eg, employment status, annual income, and respondent education) and child factors (eg, age, gender, receipt of and reason for early intervention or early childhood special education services, and functional issues [no problem, little or big problem]).

#### Young Children's Participation and Environment Measure (YC-PEM)

The 27-item YC-PEM evaluates the caregivers’ perceptions of their young child's participation in various activities that take place at home (13 items), daycare or preschool (3 items), and within the community (12 items). Upon completion of the participation items for a setting, caregivers were asked to evaluate the effect of environmental features and resources on their young child's participation (13 items for home, 16 items for daycare or preschool, and 17 items for community). A 3-point scale (3=no impact or usually helps to 1=usually makes harder) was used to assess the perceived effect of environment on participation.

The YC-PEM has three participation scales and one environment scale, which have shown fair to excellent internal consistency for the home (Cronbach alpha=.82-.96), daycare or preschool (Cronbach alpha=.67-.92), and community (Cronbach alpha=.68-.96). Test-retest reliability of the YC-PEM has also been established, using intraclass correlation coefficient (ICC) for the home (ICC=.57-.91), daycare or preschool (ICC=.31-.92), and community (ICC=.52-.94) settings. For this study, setting summary scores [12] were calculated to describe sample trends in current participation. The YC-PEM item responses were also summarized in a case report and sent electronically to caregivers to guide data collection via phone interviews.

### Data Collection

Eligible and interested participants created a user account to enroll online and complete a demographic questionnaire and the YC-PEM for the second time. Following survey completion, participants provided their contact information and availability for a semistructured phone interview to experience and provide feedback on PEM+. Before each phone interview, research staff generated a graphical YC-PEM report summarizing their responses at two time points (see Multimedia Appendix 1), which was cross-checked and sent to the participant electronically 1 to 2 days before the scheduled interview.

During the phone interview, participants were asked to review and provide feedback on the content and layout of the YC-PEM report and then try a stepwise process of building on the information in their report to specify priorities for change and to formulate goals and action plans for goal attainment. For feasibility, each participant completed the PEM+ process once during the interview. The interview guide (see Multimedia Appendix 1) was informed by formative work with community-based providers working with children and youth with developmental disabilities in a small-town community [18]. Probes in the interview guide were used to increase the likelihood of data saturation with respect to feasibility.

### Data Analysis

Sample characteristics and trends in current participation were first summarized for the total sample and subgroups (disability, no disability) using the Statistical Package for Social Sciences version 24.0 (SPSS 24.0; IBM Corp). Data were first screened via visual inspection (histogram) and normality statistics (absolute values of >2 for skewness and >7 for kurtosis) to examine whether data met assumptions of normality. Normality assumptions were also confirmed using a series of Shapiro–Wilk tests for YC-PEM participation and environment summary scores. Participation frequency scores did not deviate significantly from normal, *D* (25)=.867-.949, *P=*.09-.24. Daycare or preschool involvement and environmental support scores as well as home desire change did not deviate significantly from normal, *D* (25)=.877-.932, *P*=.10-.12. However, home and community involvement and environmental support summary scores, as well as daycare or preschool and community desire change scores, were significantly non-normal, *D* (25)= .632-.895, *P*<.001. Therefore, parametric tests were used for select subgroup comparisons only.

For main analyses pertaining to feasibility, a total of 39 phone interviews were audiotaped. The audiotaped case recordings were each reviewed independently by 2 research assistants to determine whether the recording was viable for analysis based on two criteria: (1) the recording had audible content coverage and (2) the recording contained pertinent content relative to each main question, indicating fidelity to the interview guide. Upon case review, 25 of the 39 cases (25/39, 64%) were deemed viable for analyses and therefore transcribed verbatim. A third research assistant checked each transcript with its respective digital recording to ensure accuracy before being imported into QSR International’s NVivo 11.0 for analysis.

Transcripts were content coded to the main questions in the interview guide in five phases [22]. Analytical deductive coding was used whereby relevant text (eg, words, phrases, and quotes) from each interview was sorted to a priori, which corresponded to the interview questions. Two research assistants independently coded an initial transcript that underwent code-by-code review by the principal investigator to ensure the following: (1) each excerpt included participant wording; (2) there was a match between the coded text and corresponding label; and (3) there were no missed opportunities to code interview content pertaining to one or more codes. The research assistants then proceeded to code six more transcripts, followed by another round of review by the principal investigator. Intercoder agreement was estimated to range from 80% to 95% by the third transcript.

The second research assistant proceeded to code the remaining 18 transcripts that were randomly assigned to one of four phases. Following each phase, one coded transcript was selected at random for review by the principal investigator. Discrepancies were resolved through discussion. Following analyses of all 25 cases, the second research assistant randomly selected a second coded transcript from each of the five phases for review to ensure accuracy. The principal investigator conducted a final review of all coded data to establish a final study dataset. To estimate feasibility, frequency counts were then calculated to describe PEM+ stepwise completion rates, perceived difficulty with step completion, perceived utility and report sharing preferences. Mean completion time was calculated based on start and end times for phone interviews as documented by study staff. Most findings were reported for total sample and disability subgroups, including exemplars to illustrate main findings.

To ensure credibility of the main findings, multiple researchers with different disciplinary backgrounds reviewed coded data from 13 (13/25, 52%) of the cases. To ensure dependability of main study findings, the first seven transcripts (7/25, 28%) were independently coded by 2 research staff and reviewed by the principal investigator. The principal investigator continued to review one case at random in each subsequent phase of analysis to ensure match between each code label and corresponding text. Once all the cases were analyzed, a second staff member and the principal investigator randomly selected and reviewed eight cases (8/25, 32%).

Self-reflexivity involves acknowledgement of experiences and understandings by a research member that may impact study approach and expected findings, which in turn provides authenticity and trustworthiness to the findings [22]. The first research assistant had worked at a therapeutic recreation center for children with disabilities. Her work there had exposed her to identifying participation-focused goals that do not hinge on a child’s level of independence, as well as both child and environmental strategies that parents might generate for goal attainment. During phase 1 analyses, she was closely partnering with a parent to advocate for a child, whom she had tutored, so the child could obtain interventions to address academic performance concerns that hindered his school participation. Her concurrent experience working with this family may have sensitized her to code data on parenting priorities and strategies. The second research assistant had prior experience working alongside occupational therapists, who emphasized compensatory techniques to improve patient recovery in their homes. These experiences may have sensitized her to identifying environmentally focused strategies specific to the home environment during analyses.

## Results

### Caregiver and Child Characteristics

Caregiver respondents were white mothers who were mostly married (22/25, 88%) and non-Hispanic (23/25, 92%). More than two-thirds of the families sampled were residing with multiple children in the home. As shown in Table 1, nine of the children sampled were eligible for early intervention or early childhood special education services at the time of enrollment. The most common reason for service referral was diagnosis (6/25, 24%) versus developmental delay or risk for delay. The latter indicates that a child has (or is at the risk of) an established delay in development, based on standardized developmental assessment scores, but does not have a diagnosed condition (eg, autism spectrum disorder). The most common form of rehabilitation addressing functional issues was speech and language therapy (7/25, 28%), ranging from 30 min to 3 hours per week, followed by occupational therapy (4/25, 16%) and physical therapy (2/25, 8%). There were no significant disability group differences in sociodemographic characteristics of the study sample.

**Table 1:** Sample characteristics (N=25).

Characteristic		n (%)
**Caregiver’s education^a^**		
	Some college or technical training	3 (12)
	Associate’s degree	2 (8)
	Bachelor’s degree	8 (32)
	Graduate degree	11 (44)
**Employment status**		
	Does not work for pay	12 (48)
	Part-time	7 (28)
	Full-time	6 (24)
**Household income, in US dollars ($)**		
	<$50,000	5 (20)
	$50,000-100,000	10 (40)
	>$100,000	10 (40)
**Child’s age** **(in** **years)^a,b^**		
	1-3	14 (58)
	4-5	8 (33)
	6-7	2 (8)
**Child’s gender^a^**		
	Male	13 (52)
	Female	11 (44)
**Service receipt**		
	Yes	9 (35)
	No	16 (64)
**Reported functional problems^a^**		
	Managing emotions	14 (58)
	Controlling behavior	11 (46)
	Paying attention	10 (42)

^a^indicates missing values.

^b^confirmed at the time of the interview.

Children, on average, participated once or more each week in home activities (mean=5.67, range=2.15) and daycare or preschool activities (mean=5.77, range=2.67) and once each month in community activities (mean=3.00, range=2.58). Children were somewhat to very involved in activities across home (median=4.20, interquartile range [IQR]=3.83-4.45), daycare or preschool (mean=4.6, range=1.33), and community (median=4.27, IQR=3.71-4.62) settings. Caregivers, on average, wanted their young child’s participation to change in more than half of home (13/25, 52%) and daycare or preschool (18/25, 72%) activities but not community (8/25, 32%) activities. Significant group differences between young children with and without developmental disabilities and delays were found with respect to the child’s current participation (frequency, involvement, desire change) in home and community settings (see Table 2).

**Table 2:** Disability group differences in young children’s participation and environment.

Young Children's Participation and Environment Measure (YC−PEM) scales	Disability, mean (range)	No disability, mean (range)	*t* (degrees of freedom)	*P* value
Home frequency	5.21 (1.62)	5.91 (1.85)	−3.264 (23)	.004
Home involvement^a^	3.82 (1.93)	4.25 (0.54)	-	.36
Home desire change	68.38 (84.62)	43.59 (69.23)	2.157 (23)	.04
Home environmental support^a^	82.05 (16.67)	97.44 (5.13)	-	.11
Daycare or Preschool frequency	5.50 (2.00)	5.94 (1.33)	−0.882 (8)	.40
Daycare or Preschool involvement	4.38 (1.33)	4.67 (1.00)	−0.953 (8)	.37
Daycare or Preschool desire change^a^	100.00 (33.33)	100.00 (100.00)	-	-
Daycare or Preschool environmental support	93.23 (12.50)	98.61 (2.08)	−1.780 (8)	.17
Community frequency	2.62 (2.00)	3.13 (2.25)	−1.736 (22)	.10
Community involvement^a^	3.57 (2.04)	4.38 (.53)	-	.11
Community desire change^a^	81.82 (72.73)	9.09 (27.27)	-	.03
Community environmental support^a^	84.31 (21.57)	98.04 (7.84)	-	.03

^a^indicates median (range).

### Feasibility of PEM+ for Care Plan Development

Mean PEM+ completion time as denoted by phone interview length was 38 minutes.

#### PEM+ Step 1: Identify Priorities for Change

All 25 caregivers were able to complete the first step of PEM+ in one of the two ways. Most caregivers opted to rank order the list of activities in which they had reported wanting their child’s participation to change. Only one parent opted to sort the activities according to whether the activity should be worked on now versus later.

Nearly 80% of the parents opted to rank based on importance rather than how feasible it would be to implement change to improve the child's participation in that activity. To do this, parents’ often considered the following: (1) the extent to which the activity was challenging for their child and (2) the extent to which the activity was valued by the parent. Two parents acknowledged both considerations to arrive at their respective priorities in rank order:

I guess I put...his greatest challenges first, and that the interactive and organized play he’s very rigid...[it’s] a skill that he can use in any, in every context. The socializing with friends and family, well that’s for me kind of an obvious one because we want him to take pleasure in that.

I’d rate houseguests one because I feel like she needs that interaction with people. Probably two, I would rate meal prep because I want her to be able to...help me and get that interaction with me and that bonding time with me. I would say three for the personal care because she’s still 2 and she’ll learn that as we go along.

Whereas all caregivers were able to complete this first step, 3 out of 25 caregivers (3/25, 12%) expressed some difficulty in rank ordering their priorities for change, particularly in cases where there were a large number of situations warranting change because:

...[the activities] all kind of run together.

According to another parent:

I would say it’s kind of hard. I mean, I can pick...the top one or two pretty easily, and the bottom one or two pretty easily, but the ones in the middle all kind of [blend together].

#### PEM+ Step 2: Formulate Activity-Specific Goal for Child

A total of 22 out of 25 caregivers (88%) were able to develop a goal to improve their child’s participation in their top-ranked activity or one that was sorted into the “now” category. Caregivers most often chose to focus on improving their child’s participation in a home-based activity.

Within the home setting, goals were commonly focused on improving the young child’s participation in a nondiscretionary activity such as personal care management (9/25, 36%) and cleaning up (3/25, 12%). For example, a mother of a 5-year-old boy with reported attention, communication, and sensory processing difficulties described the importance of her child’s participation in nondiscretionary activities:

...his self-care, dressing, getting clean...those are big things for him, um, to go into kindergarten.

She further elaborated on her goals for him to be more helpful in these activities:

...it could be, uh, initiating going to the bathroom on his own without being asked to go...And, uh, resting in the morning.

Similarly, a working mother described wanting her 32-month-old daughter to help clean up at home:

...when you make a mess it’s your responsibility to pick that mess up. It’s not my job to come behind you and pick it up all the time.

Whereas parents tended to describe their goals in their own words, their descriptions closely aligned with their child’s current level of participation. For example, parents described goals related to their child being more helpful or interactive in cases where their child was only somewhat involved in the activity.

#### PEM+ Step 3: Appraise Current Strategies for Goal Attainment

A total of 24 out of 25 caregivers identified strategies for goal attainment. Parent-reported strategies to improve the child’s participation focused on the child, the child’s environment, or both the child and the child’s environment.

The most common type of strategy identified by 96% (24/25) of families who had completed the YC-PEM related to modifying the child’s environment, regardless of whether or not the child had a disability. Across a broad range of home-based activities, parents described their attempts to change the physical layout of the home environment to promote the child’s engagement. For example, several parents described placing a step stool and toothbrush within reach so that the child could participate in personal care routines such as brushing teeth. Similarly, parents described setting the house up, so their child has “a place for his clothes so he knows where they go.” This type of environmental strategy extended to discretionary activities, whereby parents described placing toys and books within reach as well:

I recently like kind of reorganized his toys and like the crafts and things...and everything kind of has a place so he knows where to look and where to go. And kind of where he can see it so it's not...all like covered up. I just think he, if he can see something he can go play with...then he doesn’t...ask for a show ‘cause he has something else that he’s doing.

I mean even on a simple level...we used to keep our son's books up so he couldn’t reach them...so we moved them to his level and it’s amazing how often he goes out and picks a book and flips through it.

Apart from modifying the physical space, parents described changing the cognitive and social demands of home-based activities by setting reminders or by modeling behavior:

...having his...outfit completely laid out...in the proper way for him to be able to put it on easily without having to figure out which one’s front, which one’s back.

...reminders for her to clean up the toys.

...having the other people there and then she’s kinda watching what we’re doing and kinda copying whatever we’re doing...that’s beneficial.

Whereas the environmentally focused strategies were most common, close to one-third of families (32%) described strategies that involved having the child practice skills as preparation for engaging in the activity. This type of strategy was reported on by 56% (5/25) of caregivers raising young children with disabilities as compared with 19% (3/25) of caregivers raising young children without disabilities.

One-third of the families identified strategies for goal attainment that were directed at the child and the child’s environment. The most common type of strategy involved reinforcement strategies during mealtime. For example, 2 caregivers of children receiving services for developmental delay described offering their children desired food as an incentive for trying a new food at mealtime:

Usually if she doesn’t like what’s for dinner then she has a choice to just have cereal. She has to take a certain number of bites and then she does that and she’s still hungry and then she can just eat cereal.

So, with food, ya know, to keep out of line of sight, ya know...so, ya know, he loves yogurt. And we might do yogurt as part of lunch but yogurt doesn’t come out until he’s eaten the first part of his lunch.

#### PEM+ Step 4: Develop New Strategies for Goal Attainment

Out of 25 caregivers, 19 were able to generate new strategies for goal attainment. A vast majority of the new strategies focused on additional ways to change qualities of the child’s environment to improve participation. For example, one caregiver identified multiple strategies for allotting adequate time for cleaning up toys at home. She described allocating more time for cleaning up by pushing back the schedule, as well as by regularly going through and removing select toys to ensure that there would be a reasonable number of toys to clean up in the designated time. Another caregiver made the room darker and quieter to help her son get adequate rest, as both light and sound hindered his ability to sleep. Furthermore, a working mother of a 21-month-old girl identified ways to adjust the height of the sink and the location of her daughter’s toothbrush and toothpaste:

...an area where she could actually reach them on her own, um, and do it, she would probably be more successful. So maybe getting a table and standing next to her until she learns how to, or is tall enough to actually reach it herself.

Finally, caregivers also adjusted how children were invited to join home-based activities. For example, a mother of a 4-month-old boy could “organize the laundry in a fun way” and recalled a method where she could put her son in the laundry basket and “pull him around the house...and afterwards start folding.”

#### PEM+ Step 5: String Steps Together to Create Activity-Specific Action Plan

A total of 12 out of 25 (48%) of caregivers were able to communicate the results of the first four steps completed via phone. These caregivers could specify which activity they wanted their child to focus on first, set a goal, and identify strategies for goal attainment. However, only 4 caregivers defined a clear time frame that they considered actionable for goal attainment.

### PEM+ Completion, Perceived Utility, and Report Sharing Preferences

**Figure 2:**
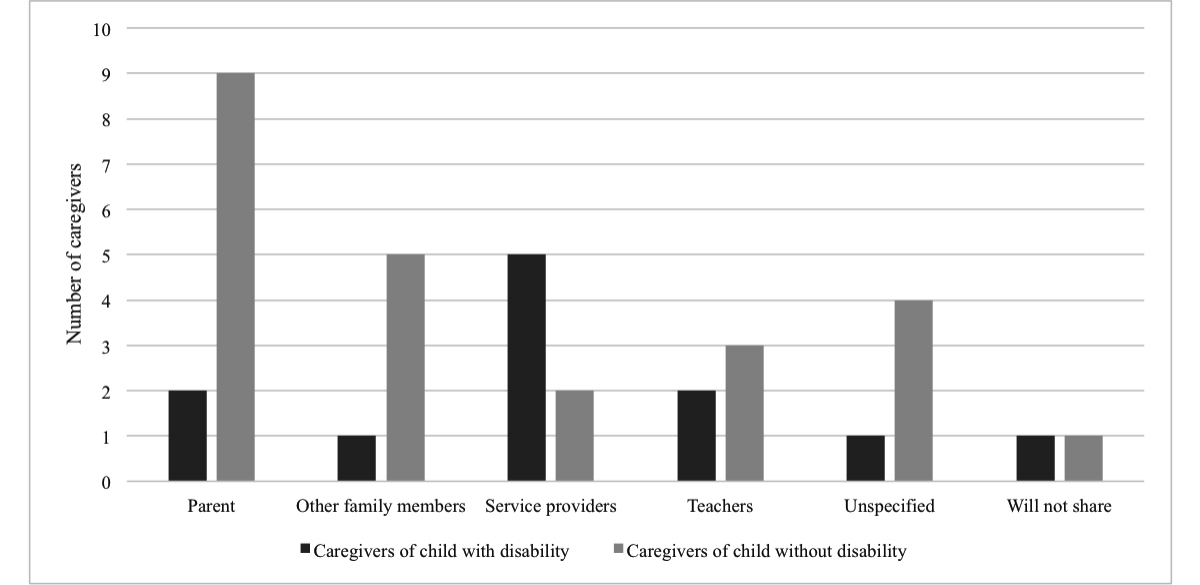
Caregiver preferences for Participation and Environment Measure (PEM) report sharing.

Mean PEM+ completion time as denoted by phone interview length was 38 min. Nearly 77% (19/25) of the users perceived the PEM+ process as useful to develop and communicate intervention priorities and strategies. Caregivers described a number of people with whom they would share their PEM report, including professionals, family, and friends (see Figure 2).

## Discussion

### Principal Findings

Patient-centered care hinges on patients having ways to collaboratively engage in designing and monitoring their care [23]. Technology may afford for more accessible provider–patient interaction [9,24] when planning and monitoring patient care. In pediatric rehabilitation, electronic PROs have emerged as one way to feasibly elicit caregiver input about the child’s functional status when the child receives care. As rehabilitation is designed to improve children’s functioning in activities of daily life, caregiver input about the child’s functional status can be used to ensure patient-centeredness in monitoring outcomes of service provision [25,26]. Pilot data on the feasibility of electronic PRO data collection within routine care suggest that they can be feasibly completed in entirety within or outside an early intervention home visit [27].

To our knowledge, this is one of the first studies to examine the feasibility of a stepwise process (PEM+) for caregivers to build on a baseline assessment of their young child’s participation and actively develop an individualized care plan for their child. Provider input helped to characterize PEM+ as a process, whereby a caregiver can establish their intervention priorities, develop goals related to each priority, and create action plans for goal attainment [18,19]. Main findings of this study suggest that 88% (22/25) or more of the caregivers sampled could engage in 4 out of the 5 parts to the PEM+ process by telephone and viewed it as an accessible way to help plan care for their young child. Several trends in stepwise completion rates and the approaches taken for step completion suggest that PEM+ can be built as an eHealth solution and are discussed.

Caregivers in this study benefited from multiple options to complete the first step of PEM+, whereby they were instructed to weigh their priorities for change and identify a top priority. Some parents expressed difficulty when rank ordering problematic activities for their child, and one parent opted to sort the activities instead. Although less common, sorting problematic activities may be a viable approach to weighing priorities for change. Sorting may be a particularly valuable approach in cases where the caregiver has identified a large number of activities in which change is desired. Patient choice is a key indicator of patient-centeredness [25] and is a common feature of eHealth technologies specific to planning care [28,29]. Both ranking and sorting options are programmable core requirements to afford for parental choice during PEM+ completion.

As parents’ expectations and priorities for change vary by context [30], future studies should examine the extent to which providing caregiver a choice to rank or sort problematic activities is useful when planning care outside a home context. In this study, caregivers commonly focused on improving their child’s participation in nondiscretionary activities at home, perhaps because home is where children spend a large amount of time [31,32], or because they have greater self-efficacy in improving conditions for participation in the home environment that they typically set up for their child and where their child receives services [15]. Alternatively, this trend in step 1 completion may have been because of the home PRO results appearing first in the PEM summary report and so were reviewed first by each caregiver during the phone interview. Regardless, generalizability of this part of the PEM+ process to out-of-home contexts warrants further study. Future studies could alter the order in which PRO results are presented in the PEM report, as well as probe to understand caregiver rationale for setting selection. Although there are fewer activity categories in the daycare or preschool section, the home and community sections of the YC-PEM PRO contain similar number of items and so would afford for stronger comparisons around trends in PEM+ step 1 completion.

Environmentally focused strategies were most commonly identified for goal attainment during PEM+ completion. This finding could be reflective of the strong environmental focus during PRO completion, as the YC-PEM involves comprehensive caregiver assessment of environmental impact on participation for each setting. Alternatively, caregivers of young children in this study may have prior experience with adapting their home environment to meet the child’s needs rather than preparing the child for the activity. This alternative hypothesis is congruent with emerging evidence about environmental impact on young children’s home participation [15,33] and the efficacy of environmentally focused interventions involving children with disabilities [34]. However, PEM+ stepwise completion rates may vary by setting. Recently, Benjamin et al [14] reported on greater caregiver knowledge and use of child-focused strategies to improve participation in daycare or preschool activities. Therefore, future studies should examine PEM+ stepwise completion rates when applied to out-of-home contexts such as the daycare or preschool setting.

New environmental strategies were also generated with phone-based intervention support for 76% (19/25) of families. These results suggest that one of the major contributions of PEM+ may be to increase parental efficacy in developing environmentally focused plans for goal attainment. This feature of PEM+ may be particularly valuable for families of young children with disabilities who receive rehabilitation services that are not functionally focused, and in turn, have increased exposure to strategies that address specific underlying impairments and prepare their child to participate in activities [35]. In this study, caregivers of young children with developmental disabilities and delays who completed PEM+ perceived their environments as providing less support for participation as compared with caregivers of young children without disabilities. These caregivers also tended to have identified child-focused strategies for goal attainment. Small sample size and lack of service use data did not allow for subgroup analyses examining the effect of service use on care plan development but warrants consideration in future studies.

Technology may enhance PEM+ functionality by affording for visualizations to help caregivers envision how to change their child’s environment. Users can also conduct queries to access data from other PEM+ users on environmental strategy use, if the content is tagged and banked by setting or activity of interest. These technological features may provide for tailored intervention support and are commonly employed in eHealth technologies for patient education [36] and emerging technology-based interventions within rehabilitation [34,37].

Trends in PEM+ completion time and report sharing lend important insight into the feasibility and use of a programmable care planning option for use by caregivers within a service context. Caregivers of children with and without developmental disabilities and delays completed PEM+ during a single phone conversation that was on average of less duration when compared with more established family assessments during a face-to-face visit [38,39]. Caregivers identified formal and informal ways to share the PEM report, although caregivers of children with disabilities most often chose to share the report with service providers who are typically tasked with soliciting for caregiver input during care plan development [40].

### Limitations

Results of this study should be considered in light of several limitations, some of which are opportunities for future study. First, approximately 34% (26/76) of families actively declined phone interviews, yet data on their reason or reasons for decline were not gathered because of feasibility. Similarly, approximately 36% (14/39) of families were excluded because of poor recording quality and fidelity. PEM+ usability testing is underway and will include tracking of enrollment trends to identify issues of sampling bias that may limit the generalizability of study results. Second, caregivers gave input on a provider-informed process [18], and PEM+ was completed by phone versus online. This level of provider involvement may have resulted in higher stepwise completion rates because of increased provider contact, as well as lower estimates of perceived difficulty and higher estimates of perceived utility due to social desirability bias. Alternatively, these higher estimates may also be due to lack of a diverse sample according to race and ethnicity and caregiver gender, or the fact that participants had completed the YC-PEM twice and were therefore familiar with its content and able to build on it during PEM+ completion. Third, we only ascertained whether and with whom the caregiver would share their PEM report, which limits our understanding of when and how they might use the information to guide decision making during rehabilitation treatment planning. Subsequent testing of a Web-based PEM+ prototype is includes access to large early intervention and early childhood agencies whose routine care is undergoing change. Hence, we anticipate enrollment of a larger and more heterogeneous sample according to race and ethnicity of the caregiver, child disability status, and family socioeconomic status to extend the generalizability of findings from this study.

### Conclusions

This study extends prior knowledge about the accessibility of electronic assessment and care planning for use by caregivers who want to consider child-focused and environmentally focused ways to improve their young child’s participation in activities of daily life. Further studies are needed to investigate how caregivers prioritize settings and whether the order in which PRO results are presented influences decision making about high priority settings. Additionally, stepwise completion rates of PEM+ when applied to out-of-home-contexts should also be examined. Work is now underway to conduct usability testing of an initial Web-based PEM+ prototype with visualizations and tiered coaching support. This testing will involve caregivers of young children aged 0 to 3 years who receive early intervention services.

## Appendix

Multimedia Appendix 1 Interview guide and sample PEM report.

## Abbreviations

eHealth: electronic health
ICC: intraclass correlation coefficient
IQR: interquartile range
PRO: patient-reported outcome
RBI: routines-based interview
PEM: participation and environment measure
PEM+: participation and environment measure-plus
YC-PEM: young children’s participation and environment measure

## References

[1] American Occupational Therapy Association (2017). Occupational therapy practice framework: domain and process (3rd edition). Am J Occup Ther.

[2] World Health Organization (WHO) (2007). Apps.who.int.

[3] Khetani MA, Richardson Z, McManus BM (2017). Social disparities in early intervention service use and provider-reported outcomes. J Dev Behav Pediatr.

[4] Imms C, Adair B, Keen D, Ullenhag A, Rosenbaum P, Granlund M (2016). 'Participation': a systematic review of language, definitions, and constructs used in intervention research with children with disabilities. Dev Med Child Neurol.

[5] Imms C, Granlund M, Wilson PH, Steenbergen B, Rosenbaum PL, Gordon AM (2017). Participation, both a means and an end: a conceptual analysis of processes and outcomes in childhood disability. Dev Med Child Neurol.

[6] Amtmann D, Cook KF, Johnson KL, Cella D (2011). The PROMIS initiative: involvement of rehabilitation stakeholders in development and examples of applications in rehabilitation research. Arch Phys Med Rehabil.

[7] Hebbeler K, Spiker D, Bailey D, Scarborough A, Mallik S, Simeonsson R, Singer M, Nelson L Sri.

[8] McWilliam R (2009). Iidc.indiana.

[9] Wang S, Blazer D, Hoenig H (2016). Can eHealth technology enhance the patient-provider relationship in rehabilitation?. Arch Phys Med Rehabil.

[10] Khetani MA, Cohn ES, Orsmond GI, Law MC, Coster WJ (2013). Parent perspectives of participation in home and community activities when receiving part C early intervention services. Topics Early Child Spec Educ.

[11] Khetani MA, Orsmond G, Cohn ES, Law M, Coster W (2012). Correlates of community participation among families transitioning from Part C early intervention services. OTJR (Thorofare N J).

[12] Khetani M, Graham JE, Alvord C (2013). Community participation patterns among preschool-aged children who have received Part C early intervention services. Child Care Health Dev.

[13] Khetani MA, Graham JE, Davies PL, Law MC, Simeonsson RJ (2015). Psychometric properties of the Young Children's Participation and Environment Measure. Arch Phys Med Rehabil.

[14] Benjamin TE, Lucas-Thompson RG, Little LM, Davies PL, Khetani MA (2017). Participation in early childhood educational environments for young children with and without developmental disabilities and delays: a mixed methods study. Phys Occup Ther Pediatr.

[15] Albrecht EC, Khetani MA (2017). Environmental impact on young children's participation in home-based activities. Dev Med Child Neurol.

[16] Commondataelements.ninds.nih.

[17] Lim CY, Law M, Khetani MA, Pollock N, Rosenbaum P (2016). Establishing the cultural equivalence of the Young Children's Participation and Environment Measure (YC-PEM) for use in Singapore. Phys Occup Ther Pediatr.

[18] Khetani MA, Cliff AB, Schelly C, Daunhauer L, Anaby D (2014). Decisional support algorithm for collaborative care planning using the Participation and Environment Measure for Children and Youth (PEM-CY): a mixed methods study. Phys Occup Ther Pediatr.

[19] Arestad K, Nale LN, Albrecht E, Khetani MA (2015). Caregiver input to develop a web-based care planning guide focused on young children's participation. Arch Phys Med Rehabil.

[20] Gleason M, Arestad K, Nale L, Khetani MA (2015). Parent input informing the development of an intervention planning guide to improve child and youth participation. Am J Occup Ther.

[21] Constand MK, MacDermid JC, Dal Bello-Haas V, Law M (2014). Scoping review of patient-centered care approaches in healthcare. BMC Health Serv Res.

[22] Creswell JW (2007). Qualitative Inquiry and Research Design: Choosing Among Five Approaches. 2nd edition.

[23] Gpo.

[24] Ellis K, Kent M (2011). Disability and New Media.

[25] Mroz TM, Pitonyak JS, Fogelberg D, Leland NE (2015). Client centeredness and health reform: key issues for occupational therapy. Am J of Occup Ther.

[26] Coster WJ, Khetani MA (2008). Measuring participation of children with disabilities: issues and challenges. Disabil Rehabil.

[27] Nale L, Arestad K, Khetani MA, Charlifue-Smith R, Rosenberg C, Richardson Z, McManus B (2016). Feasibility of technology-based functional outcomes data gathering in early intervention. Arch Phy Med Rehabil.

[28] Uhler LM, Pérez Figueroa RE, Dickson M, McCullagh L, Kushniruk A, Monkman H, Witteman HO, Hajizadeh N (2015). InformedTogether: usability evaluation of a web-based decision aid to facilitate shared advance care planning for severe chronic obstructive pulmonary disease. JMIR Hum Factors.

[29] Cardona-Morrell M, Benfatti-Olivato G, Jansen J, Turner RM, Fajardo-Pulido D, Hillman K (2017). A systematic review of effectiveness of decision aids to assist older patients at the end of life. Patient Educ Couns.

[30] Tamis-LeMonda CS, Way N, Hughes D, Yoshikawa H, Kalman RK, Niwa EY (2008). Parents' goals for children: the dynamic coexistence of individualism and collectivism in cultures and individuals. Soc Dev.

[31] Law M, Anaby D, Teplicky R, Khetani MA, Coster W, Bedell GM (2013). Participation in the home environment among children and youth with and without disabilities. Br J Occup Ther.

[32] Khetani MA, Graham JE, Davies PL, Law MC, Simeonsson RJ (2015). Psychometric properties of the Young Children's Participation and Environment Measure. Arch Phys Med Rehabil.

[33] Law MC, Darrah J, Pollack N, Wilson B, Russell DJ, Walter SD, Rosenbaum P, Galuppi B (2011). Focus on function: a cluster, randomized controlled trial comparing child versus context-focused intervention for young children with cerebral palsy. Dev Med Child Neurol.

[34] Anaby D, Law M, Teplicky R, Turner L (2015). Focusing on the environment to improve youth participation: experiences and perspectives of occupational therapists. Int J Environ Res Public Health.

[35] Workgroup on Principles and Practices in Natural Environments Ectacenter.

[36] Wicks P, Stamford J, Grootenhuis MA, Haverman L, Ahmed S (2014). Innovations in e-health. Qual Life Res.

[37] Wade SL, Walz NC, Carey J, Williams KM, Cass J, Herren L, Mark E, Yeates KO (2010). A randomized trial of teen online problem solving for improving executive function deficits following pediatric traumatic brain injury. J Head Trauma Rehabil.

[38] McWilliam RA, Casey AM, Sims J (2009). The routines-based interview: a method for gathering information and assessing needs. Infants Young Child.

[39] Ziviani J, Poulsen A, Cuskelly M (2015). Goal Setting and Motivation in Therapy: Engaging Children and Parents.

[40] The American Occupational Therapy Association Aota.

