# Correction: Caregiver Input to Optimize the Design of a Pediatric Care Planning Guide for Rehabilitation: Descriptive Study

**DOI:** 10.2196/rehab.9580

**Published:** 2017-12-21

**Authors:** Mary A Khetani, Heather K Lim, Marya E Corden

**Affiliations:** ^1^ Children's Participation in Environment Research Lab Department of Occupational Therapy University of Illinois at Chicago Chicago, IL United States

The article “Caregiver Input to Optimize the Design of a Pediatric Care Planning Guide for Rehabilitation: Descriptive Study” (JMIR Rehabil Assis Technol 2017; 4(2):e10) had several errors. These errors have been corrected as follows:

**Introduction:**

In the “Background” subsection, fifth paragraph, the text should read: “Participation and Environment Measure Plus (PEM+) is a guide that is compatible with the YC-PEM and may expedite care plan development and strengthen a patient’s engagement in discussions and decisions about their values, needs, and desires that shape meaningful care (ie, patient-centered care).” This sentence was corrected to be grammatically correct.

**Results:**

In the “PEM+ Step 3: Appraise Current Strategies for Goal Attainment” subsection, fourth paragraph, the text should read: “This type of strategy was reported on by 56% (5/9) of caregivers raising young children with disabilities as compared with 19% (3/16) of caregivers raising young children without disabilities.” This was corrected to denote the correct denominator for the corresponding subgroup.

The text of the last subsection should read: “PEM+ Completion Time, Perceived Utility, and Report Sharing Preferences”. This header is corrected to accurately reflect the content of this subsection in results.

**Discussion:**

In the “Principal Findings” subsection, second paragraph, the text should read: “Provider and caregiver input helped to characterize PEM+ as a process, whereby a caregiver can establish their intervention priorities, develop goals related to each priority, and create action plans for goal attainment [18,19].” This sentence was corrected so that it aligns with the two references that are listed as supporting this statement.

In the “Principal Findings” subsection, fourth paragraph, the text should read: “Since parents’ expectations and priorities for change vary by context [30], future studies should examine whether providing caregivers with choice about ranking or sorting problematic activities helps in planning care outside of the home context.” This sentence was corrected as it contained a typographical error.

In the “Limitations” subsection, the text should read: “Subsequent testing of a Web-based PEM+ prototype is underway and includes access to large early intervention and early childhood agencies whose routine care is undergoing change.”  This sentence was corrected as it contained a typographical error. 

The corrected article will appear in the online version of the paper on the JMIR website on December 21, 2017, together with the publication of this correction notice. Because this was made after submission to PubMed or Pubmed Central and other full-text repositories, the corrected article also has been re-submitted to those repositories.

