# Supplementary material for: Caregiver Input to Optimize the Design of a Pediatric Care Planning Guide for Rehabilitation: Descriptive Study
Source: JMIR Rehabil Assist Technol. 2017 Oct 24;4(2):e10. doi: 10.2196/rehab.7566 (PMC5676028; doi:10.2196/rehab.7566)
Supplement: Multimedia Appendix 1 [file rehab_v4i2e10_app1.pdf]

YC-PEM Follow-up Interview Guide

1. Introduction & Purpose of Study Section
   - Thank participant for being a continued part of the study.
   - Disclose the length of time expected for the conversation (30 min), and that it is being recorded.
   - Explain the goal for the conversation: To review the results of the follow up YC-PEM assessment in light of last year's results, and to ask for feedback on the report used for this purpose.
     - EXAMPLE: *"Last year, you completed the YC-PEM and we asked you to do it again this year. Our conversation today will review the changes in your child's participation, and we also want to try out a way to summarize this evaluation through the report we sent you. We'd love your feedback on the report to help us develop it for use with parents in the future."*
2. Orient participant to the report
   - Take parent through the report in the order. Report begins with three pages of summary charts of frequency and involvement in activities in home, school/daycare, and community.
     - *It is important to begin this conversation by pointing out positive aspects of child's participation to foster rapport.
     - This chart will contain data from both times the parent has taken the YC-PEM. Walk them through the changes that have occurred between the two evaluations.
     - EXAMPLE: *"Turn to the second page of the report. This page summarizes the information you shared about your child's participation at home. On this page you should see two graphs. The graph on the left shows how often your child does the activities, and the graph on the right shows how involved (s)he is in those activities. For example, it looks like (s)he does ________ once or more daily, and when (s)he does, (s)he's very involved."*[and so on for each of the three settings]
   - Guide parent through tables that list activities that they reported desiring change.

- EXAMPLE: *"The next section begins on page 5, with a table that lists the things that you said you'd like to see change. There's a table for each setting, with three columns, showing the activity, the type of change you want to see, and then some of the strategies you're already using to help with that activity."*

3. Choose a setting to focus on first
   - Guide parent to decide which of the three settings they find needs the most urgent intervention, in their opinion.
     - EXAMPLE: *"When you look at the three settings, home, daycare/preschool, and community, do any of these three seem more important for you to address before the others?"*
     - PROBE: (if parent expresses difficulty choosing with importance) *"Do any of these settings seem more feasible to work on?"*
4. Ask parent to read through the activities where change is desired, listed under their chosen setting, and put them in rank order of importance for intervention.
   - If parent has difficulty with importance, ask them to consider listing by feasibility, or to sort them in to categories of most, somewhat and least important and/or feasible.
     - EXAMPLE: *"Okay, we'll concentrate on the _______ setting for this conversation. Next, I'd like you to read through the activities you listed as wanting to change in the _______ setting, and then rank them in order of most important to address."*
     - PROBE (if parent expresses difficulty with importance ranking): *"What if we ranked them in order of which is the most feasible to address right away?" OR "Do you think you can categorize these into Most Important, Somewhat Important, and Least Important? Then we can concentrate just on the activities that you think are the very most important to discuss further."*
5. Summarize conversation to this point: *"So, to recap, we've decided to discuss the ________ setting, and of the activities listed there, you would like to address __________ first.*
6. Take parent back to the graphs and discuss the changes desired and confirm and clarify that they match the assessment data (i.e., a response on the table for desire for change that states that a parent would like the child to do an activity "more often" is summarized on the

chart as being done "more than once daily," may require further clarification to understand what exactly the parent wants to see change.).

- EXAMPLE: *"You've listed the ______ activity as the most important. Let's take a look back at the summary page with the graphs on it to look a little closer at your child's participation in that activity. Last year, you reported that your child did this activity [frequency], and this year (s)he does it [frequency], so you've experienced [change or no change in frequency]. (S)he was [involvement] last year, and this year you report that (s)he is [involvement] when (s)he participates in this activity, so you've seen [change or no change in involvement]. Do you agree with that summary?"*

7. Point out strategies that the parent is already using (listed to the right of the activities).
   - PROBE: *"On the table where you list the changes you desire, you also listed some strategies that you are already using to help with this activity. For this activity, you listed __________. What do you think is the focus of this strategy? Is it about changing your child, your child's environment, or both?"*
8. Ask parent about what in the environment helps and hinders the child in doing this activity.
   - If parent has any difficulty with this, use data on the environment tables in the report to further discussion, and sometimes clarification that the environment can be more than just the built structures, and can include sensory qualities, attitudes of others, etc."
     - PROBE: *"Does the environment help your child participate in this activity? Can you make changes to the environment that would make it easier for your child to participate in this activity?"*
9. Finalize action plan:
   - The first activity you want to work on first is ______________________.
   - Your goal is to _____________________ by _______________.
   - Things that you will continue doing to help you reach this goal are _________________________.
   - Some things that need to be addressed to reach this goal are ________________________.

10. Seek feedback on process: Ask parent what was helpful about this process, what was difficult/easy, and if they have any ideas about how to improve this process. Also seek feedback on how to disseminate information in the future to parents in this study about how to be/stay involved with the research.
    - PROBE: *"That's our process! The idea is that we would do this for other activities too. How was this process for you? Was anything particularly difficult for you? What did you think of comparing the two years' worth of information? Was there anything especially difficult about this? Did this process help bring your attention to the environment and how it effects participation?"*
11. Confirm mailing address.

Participation Profile

Date of 2nd assessment: 12/12/2014

The foundation for lifelong health is built during the first 5 years of your child's life. When young children participate in activities, they can feel healthy, learn new skills, and have fun. Young children participate with important people in their life like you, their parent or primary caregiver.

This report has your answers to the questions that we asked you, so that you can see:

1. How your child participates in activities (last year and this year);
2. How you want your child's participation to change;
3. What you already do to help your child participate;
4. Things in the environment that help or make it harder for your child to participate.

Home Participation Profile

This diagram shows **how often** your child participates in home activities.
(1= never, 2= once in the last 4 months, 3= few times in the last 4 months, 4= once in the last month, 5= few times in the last month, 6= once each week, 7= few times each week, 8= once or more each day)

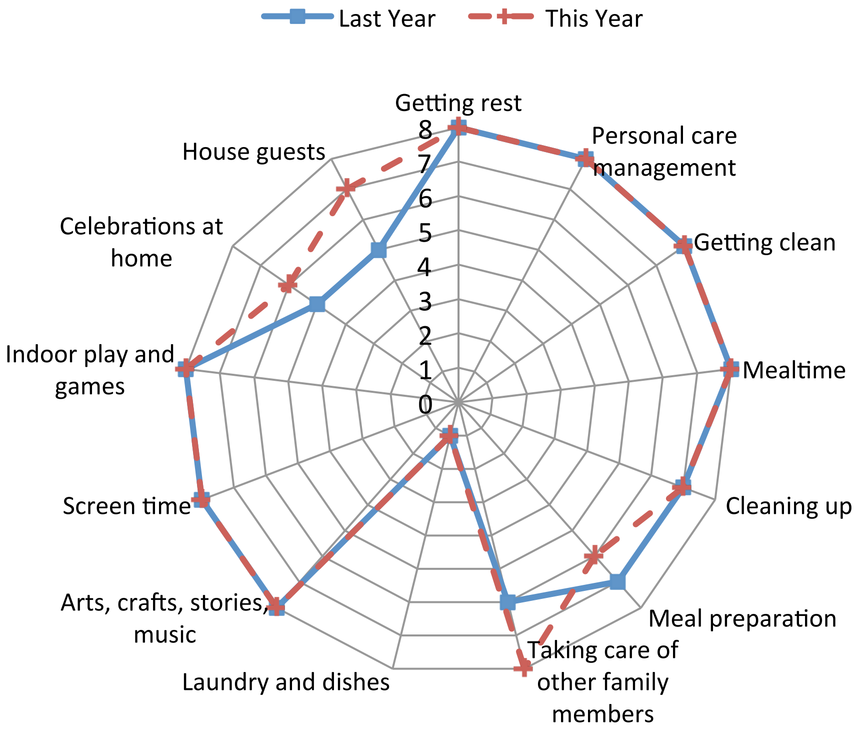

This diagram shows **how involved** your child is when participating in home activities.
(1= not very involved, 3= somewhat involved, 5= very involved)

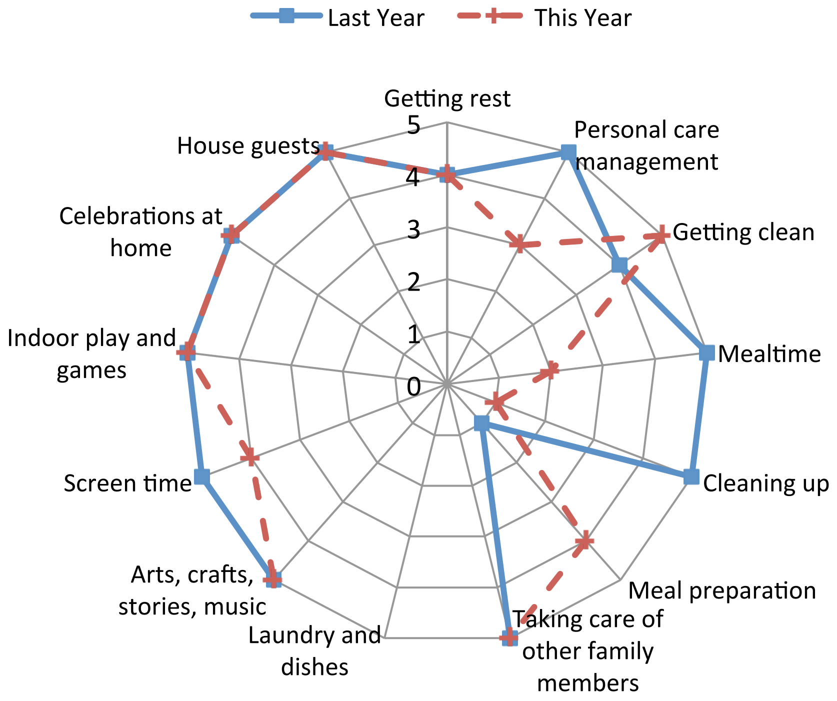

Daycare/Preschool Participation Profile

This diagram shows **how often** your child participates in daycare/preschool activities.
(1= never, 2= once in the last 4 months, 3= few times in the last 4 months, 4= once in the last month, 5= few times in the last month, 6= once each week, 7= few times each week, 8= once or more each day)

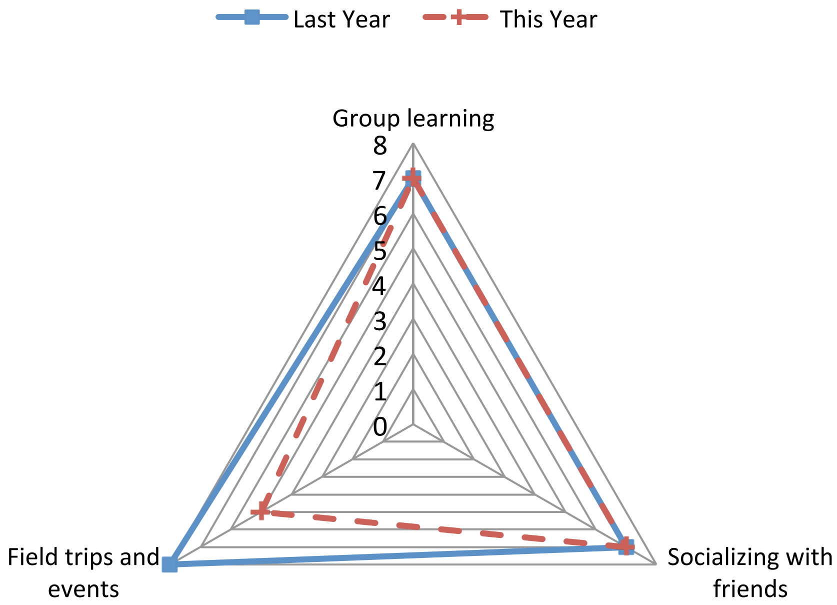

This diagram shows **how involved** your child is when participating in daycare/preschool activities.
(1= not very involved, 3= somewhat involved, 5= very involved)

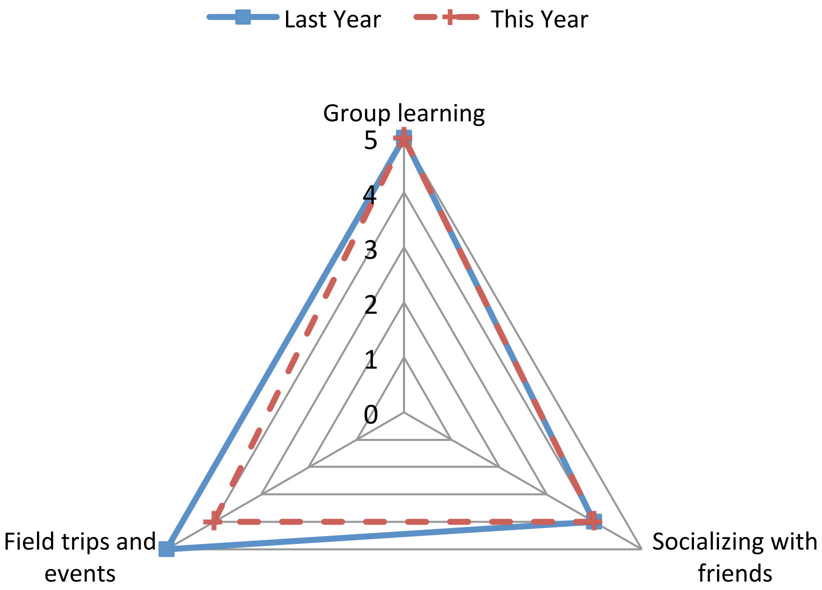

Community Participation Profile

This diagram shows **how often** your child participates in community activities.
(1= never, 2= once in the last 4 months, 3= few times in the last 4 months, 4= once in the last month, 5= few times in the last month, 6= once each week, 7= few times each week, 8= once or more each day)

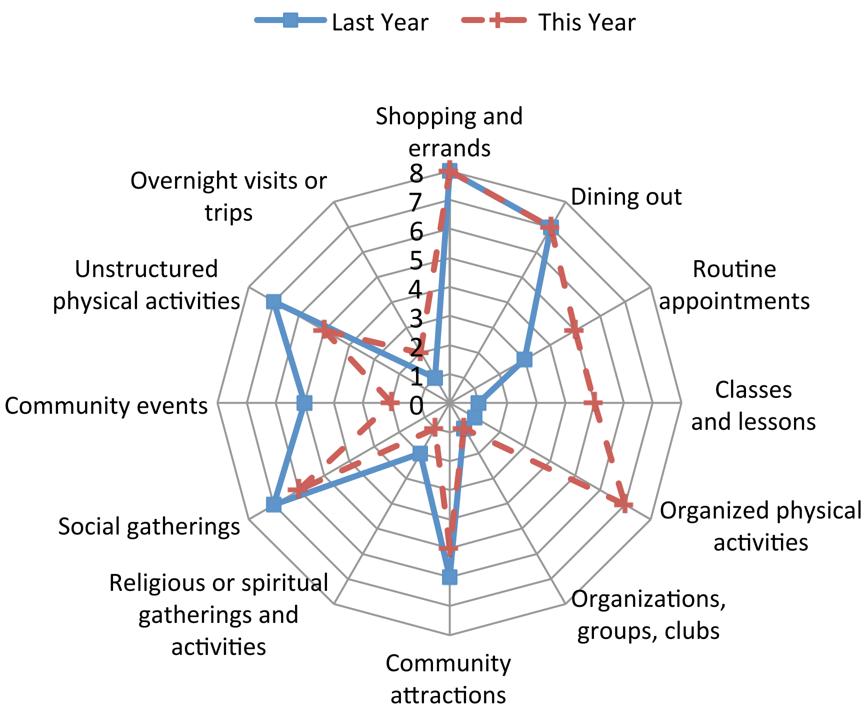

This diagram shows **how involved** your child is when participating in community activities.
(1= not very involved, 3= somewhat involved, 5= very involved)

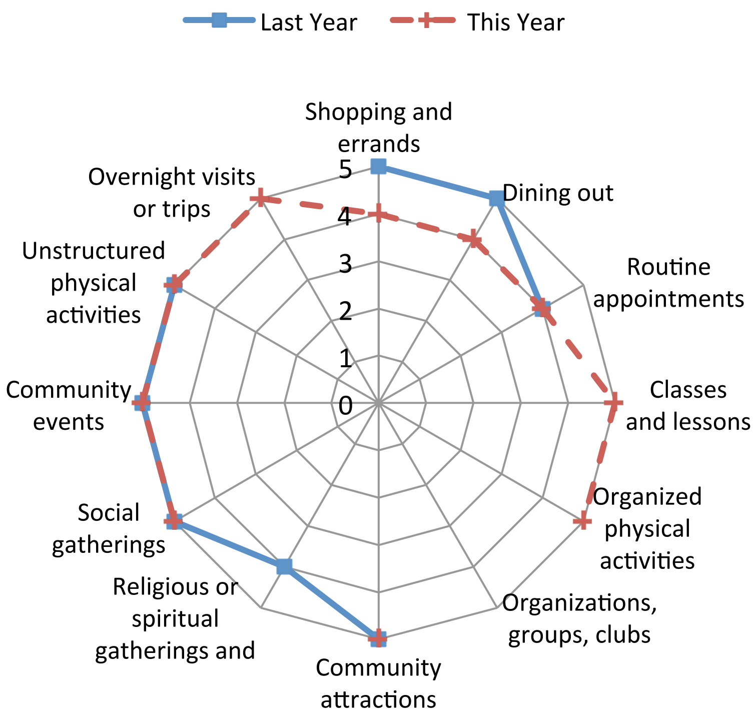

WHAT I WANT TO CHANGE ABOUT MY CHILD'S PARTICIPATION

Here are some areas in which I want my child's participation to change and what I have done to help my child participate in those activities.

HOME

Change Desired: 1= no change desired; 2= do more often; 3= do less often; 4= be more helpful; 5= be more interactive; 6= a broader variety.

Activities		Change Desired?	My strategies
Basic Care Routines	Getting rest	Yes, be more interactive	1. When she hasn't had a nap during the day she is more ready for sleep at night. If I lay down with her she will sleep or nap more easily<br>2. She will dress herself if there is somewhere she wants to go. I don't expect her to brush her own teeth yet She prefers to have help in the bathroom even though I know she doesn't get help at school<br>3. When she can help with meal preparation, she is better about eating. The better she sleeps, the better she is to eat breakfast
	Personal care management	Yes, be more interactive	
	Getting clean	No	
	Mealtime	Yes, be more interactive	
Household Chores	Cleaning up	Yes, 2 and 6	1. When her brothers are also helping put away toys, she will help better.
	Meal preparation	No	
	Taking care of other family members	No	
	Laundry and dishes	No	
Interactive and organized play	Arts, crafts, stories, music	No	1. N/A
	Screen time	No	
	Indoor play and games	No	

Socializing with friends and family	Celebrations at home	No	1. N/A
	House guests	No	

Strategies
1. She is included as part of the family and expected to participate.<br>2. She has lots of age appropriate toys that she enjoys playing with<br>3. Her brothers are expected to actively include her

DAYCARE/PRESCHOOL

Change Desired: 1= no change desired; 2= do more often; 3= do less often; 4= be more helpful; 5= be more interactive; 6= a broader variety.

Activities		Change Desired	My strategies
Educational Programming	Group Learning	No	1. She is usually more interested in socializing than in eating a meal where friends are involved. I would like to see her eat a little better before going into full play.
	Socializing with friends	Yes, be more interactive	
	Field trip and events	No	

Strategies
1. I make sure that she wears comfortable clothes and shoes<br>2. I make sure that she arrives on time<br>3. I provide snacks and anything else requested by school.

COMMUNITY

Change Desired: 1= no change desired; 2= do more often; 3= do less often; 4= be more helpful; 5= be more interactive; 6= a broader variety.

Activities		Change Desired	My strategies
Neighborhood and community outings	Shopping and errands	No	1. If she is hungry perhaps she will eat better.<br>2. If her brothers are eating better she will also.
	Dining out	Yes, be more interactive	
	Routine appointments	No	
Classes and groups	Classes and lessons	No	1. N/A
	Organized physical activities	No	
	Organizations, groups, clubs	No	
Community-sponsored activities	Community attractions	No	1. N/A
	Religious or spiritual gatherings and activities	No	
	Social gatherings	No	
	Community events	No	
Recreational activities and trips	Unstructured physical activities	No	1. N/A
	Overnight visits or trips	No	

Strategies
1. There are lots of variety of things to do.

ENVIRONMENT

Here are some things in the environment that I think **help my child** participate in activities:

Home	Daycare/Preschool	Community
The physical layout	The physical layout	Physical layout or amount of space
Sensory qualities of home	Sensory qualities	The attitudes and actions of others
Supplies	The physical demands of activities	Child's relationships with peers
Information	The cognitive demands of activities	Outside weather conditions
Time	The social demands of activities	The safety of the community
Money	Child's relationships with peers	Access to personal transportation
	The attitudes and actions of others	Equipment or supplies
	School-related policies and procedures	Information
	Access to personal transportation	Time
	Supplies	Money
	Information	
	Time	
	Money	
	Outside weather conditions	

Here are things in the environment that I think **make it harder** for my child to participate in activities:

Home	Daycare/Preschool	Community
None reported	None reported	None reported

SO WHAT'S NEXT?

We hope that you will share this report with your family, friends, colleagues, and others with whom you want to share this information (e.g., teachers, health professionals, coaches). We would also be happy to review this report with you by phone, answer your questions, and brainstorm together how you could use this information.

Children's Participation and Environment Research Laboratory

I Email: ycpemsurvey@colostate.edu I I Phone: 970.491.1795 I I Website: http://www.cperl.colostate.edu I
